# Chemical Composition, Biological Activity, and Potential Uses of Oregano (*Origanum vulgare* L.) and Oregano Essential Oil

**DOI:** 10.3390/ph18020267

**Published:** 2025-02-18

**Authors:** Renata Nurzyńska-Wierdak, Magdalena Walasek-Janusz

**Affiliations:** Department of Vegetable and Herb Crops, Faculty of Horticulture and Landscape Architecture, University of Life Sciences in Lublin, Doświadczalna 50a Street, 20-280 Lublin, Poland; magdalena.walasek@up.lublin.pl

**Keywords:** Lamiaceae, medicinal and aromatic plants, natural antibacterial agents, antioxidant activity

## Abstract

Medicinal aromatic plants (MAPs) are a rich and diverse source of traditional and modern medicines. Aromatic plants contain valuable essential oils that exhibit numerous biological activities. Essential oils are used in pharmaceutical production, cosmetics, and food preservation to ensure the microbiological stability of products. Plants from the Lamiaceae family, including *Origanum vulgare* L., are a source of raw materials with antimicrobial and antioxidant properties, and they can be utilized in the production of new drugs and other bioproducts. Oregano is an example of a plant with great potential, known for its traditional health-related and culinary applications and its growing significance in the production of medicines, cosmetics, antiseptics, and preservatives. This work aims to consolidate the current research results on the occurrence, acquisition, use, and medicinal and dietary value of common oregano and oregano essential oil. The obtained results indicate that oregano is a valuable medicinal and culinary plant, serving as a source of natural antiseptics and protective agents. Oregano essential oil, rich in thymol and carvacrol, has a number of health-promoting properties. These compounds (also present in extracts) exhibit significant antioxidant, anti-inflammatory, antiangiogenic, anticancer, and antimicrobial activities. The research findings highlight the promising role of these compounds as potential structures in the search for new antimicrobial and antibiofilm agents.

## 1. Introduction

Medicinal aromatic plants (MAPs) are a rich and diverse source of traditional and modern medicines and cosmetics. Among them are wild plants and plants grown in herbal plantations, known for their traditional medicinal and culinary uses and processing. The unique aroma of MAPs is associated with the presence of essential oils (EOs), volatile substances of a complex chemical nature [1]. EOs contain terpene compounds, such as alcohol derivatives (geraniol, α-bisabolol), ketones (menthone, p-vetivone), aldehydes (citronellal, sinensal), esters (γ-terpinyl acetate, cedryl acetate), and phenols (thymol), as well as non-terpene compounds and aromatic phenylpropane derivatives, including eugenol, cinnamaldehyde, and safrole. The primary metabolic precursors of terpenoids and phenylpropanoids are compounds produced by various biosynthetic pathways/routes [2]. EOs are secondary metabolites, plant secretions that can perform essential functions in the plant body: they can protect against viruses, bacteria, fungi, insects, and herbivores, as well as attract potential pollinators [3]. The biological properties of EOs are generally associated with their significant constituents, which are present at 20–70% [4]. EOs are used in pharmaceutical production, cosmetics, and food preservation to ensure the microbiological stability of products. In aromatherapy, their valuable therapeutic properties are utilized, including their antiinflammatory, antimicrobial, antioxidant, sedative, analgesic, and antistress properties [5]. In addition to EOs, other bioactive components of MAPs, including active polyphenols [1,6,7], also have potential applications in medicine and pharmacy.

The Lamiaceae family includes many MAP species valued for their use in medicine, food, and cosmetics. It is one of the most essential plant groups producing EOs with antioxidant and antimicrobial properties. Lamiaceae plants have been proven to be a source of raw material with antimalarial [8], antimicrobial [9], and antioxidant [10] properties and can be used in the production of new drugs and other bioproducts such as flavorings, cosmetics, or preservatives [11]. One of the better-known members of the Lamiaceae family is the genus Origanum, which includes Mediterranean plant species traditionally used as spices and medicines.

## 2. Materials and Methods

Scientific information was obtained by searching the resources of available databases (CABI, EBSCO, Science Direct, Find Articles, JCR, Medline, Oxford Journals, Scopus, Springer, Web of Science, Wiley) to review and consolidate the results of current research on the occurrence, acquisition, use, and dietary and medicinal value of common oregano (*Origanum vulgare* L., Lamiaceae) plants. A classical methodology consisting of keyword generation, systematic search, cataloguing, and subsequent discussion of relevant scientific information was used. The conclusion summarizes recent critical literature data and outlines further research perspectives.

## 3. Results

### 3.1. Species Description, Occurrence, Traditional Use

The genus Origanum (Lamiaceae) is divided into ten sections comprising 43 species, 6 subspecies, 3 cultivars, and 18 natural hybrids, including annuals, perennials, and shrubs, which are commonly found in the Mediterranean, Euro–Siberian, and Iranian–Siberian regions [12]. Most species in the genus (about 75%) are found in the western Mediterranean area, with 16 considered endemic to Turkey [13]. Wild oregano species are found at different altitudes, from coastal to mountainous areas, on different soils, have high light requirements, and can withstand relatively low temperatures. The more important genera of oregano include Greek oregano (*O. vulgare* L. ssp. *hirtum*), Turkish oregano (*O. onites* L.), common oregano (*O. vulgare* L. ssp. *vulgare*), and *O. vulgare* L. ssp. *viridulum* [14]. Within the species *O. vulgare* L., there is considerable morphological and chemical diversity. Studies on the systematic diversity, genetic diversity, and identification of the Origanum species have mainly focused on morphological characteristics and essential oil composition. Recently, molecular markers have been used to detect DNA polymorphism between/among populations and to identify phylogenetic relationships [15]. Notably, selecting 70 individual plant clones from 59 populations of oregano of different origins, carried out in France according to macromorphological criteria, showed a lack of correlation between morphological and chemical structure [16].

*O. vulgare* L. (common oregano, wild marjoram, Spanish marjoram) is probably the best-known species of the genus Origanum and has uses in traditional medicine and as a culinary spice. The name Origanum was first used by Hippocrates (460–370 BC), comes from the Greek words for mountain (oros) and joy (ganos), and can be translated as ‘joy of the mountains’ [15]. *O. vulgare* is a perennial plant growing to more than 100 cm. It forms an erect, top-branching stem, sometimes reddish in color (Photo S1). The leaves of oregano are petioled, broadly ovate to elliptically lanceolate, entire or finely sawn, and arranged oppositely. The plant produces numerous small, pink, pink-lilac or white flowers, gathered in pinnate sub-buds (Photo S2), containing quite a lot of nectar. Oregano’s pollinating insects are mainly bees and bumblebees; numerous butterfly species also visit oregano flowers [15,17]. The flowering period of oregano is long and lasts from June to September; flowering is abundant, and the plant is highly decorative during this time. The characteristic feature of this species is the glandular hairiness of the entire plant and the intense, pleasant, herbaceous, and spicy fragrance reminiscent of thyme. Oregano is an oil plant, and the thymol present in the oil causes odor [18,19]. The raw herbal material is the oregano herb (*Origani herba*), collected from crops and natural sites at the beginning of plant flowering when it contains the essential oil [20,21]. Skoufogianni et al. [14] predict an increase in the cultivation and use of *O. vulgare* L. in Greece and the entire Mediterranean region in the near future, due to its high medicinal, economic, and environmental value.

The oregano herb is used as a spice to produce oregano essential oil (OEO) and oregano extracts (OEX). These products have antiseptic and expectorant effects, as well as a cholagogue effect. They are used as remedies to improve digestion, for mouth and throat rinses, for inhalations for upper respiratory tract inflammation, and for medicinal baths [22,23]. OEO and OEX have been described as promising natural food preservatives and as agents beneficial for gastrointestinal function, particularly in inflammation [24]. Fresh and dried oregano herb is a valued seasoning for various dishes (soups, sauces, meats, vegetables, preserves) and an ingredient in aromatic spice blends.

### 3.2. Chemical Composition of Oregano Essential Oil and Its Variability

The primary and secondary metabolites produced by oregano plants are crucial for biological activity. The primary biologically active substance is OEO, which has a diverse chemical composition. The chemical composition of OEO is largely caused by genetic and environmental variability. *O. vulgare* plants can synthesize at least nine essential oil chemotypes, the predominant components being thymol, sabinene, *trans*- and *cis*- sabinene hydrate, germacrene D, β-caryophyllene, (Z)-β-ocimen, and (E)-β-ocimen [16,18,19,25,26] (Table 1). Russo et al. [27] presented 56 EO components in plants growing wild in southern Italy, while D’Antuono et al. [28], characterizing wild Italian oregano populations, identified 64 EO components. Based on phenolic content, the authors identified four chemotypes of Calabrian oregano: thymol (the most common), carvacrol, thymol–carvacrol, and carvacrol–thymol. On the other hand, Macedonian OEO was characterized by trace amounts of thymol and the absence of carvacrol [29]. Wild populations of oregano in Montenegro accumulated between 0.7 and 1.2% (*O. vulgare* subsp. *vulgare*) and 3% EOs (*O*. *vulgare* subsp. *hirtum*) [30]. Andi et al. [31] identified four chemotypes of carvacrol, sabinene, caryophyllene oxide, and linalyl acetate in native Iranian populations of *O. vulgare* ssp. *vulgare*. Verma et al. [18], analyzing the EOs of 17 populations of oregano from the western Himalayas, found 0.07 to 0.80% EOs, which included monoterpene hydrocarbons (2.85–69.2%), oxidized hydrocarbons (from trace amounts to 58.57%), sesquiterpene hydrocarbons (0.29–42.14%), oxidized sesquiterpenes (from trace amounts to 24.44%), phenolic monoterpenoids (21.10–69.49%), and aliphatic alcohols (from trace amounts to 2.71%).

**Figure 1:**
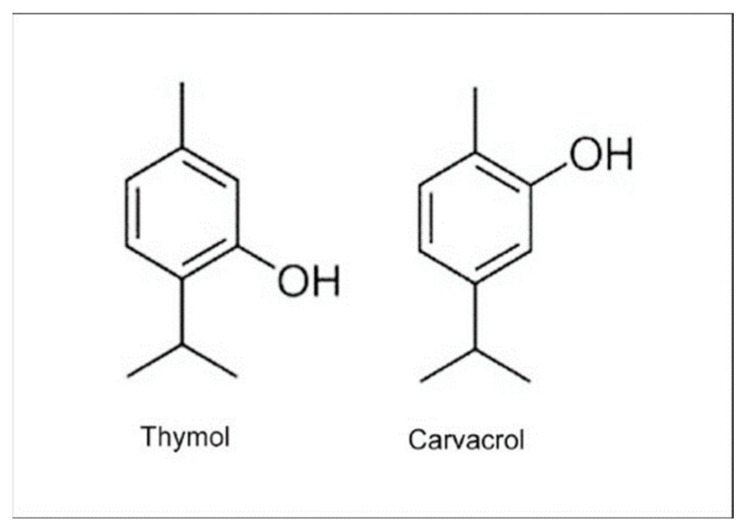
Structure of thymol and carvacrol.

The results cited here demonstrate the considerable variation in OEO’s content and chemical composition caused by genetic, ontogenetic, and environmental variation, with important implications for using OEO and OEX in medicine. The most significant differences concern the content of the highly active phenolic compounds thymol and carvacrol (Figure 1), whose proportion in OEO is variable. Both compounds exhibit broad biological activities, including antispasmodic, antiinflammatory, anticancer, antimicrobial and potent antioxidant activity [33,37,38]. Carvacrol and thymol are potent antimicrobial agents against Gram-positive and Gram-negative bacteria. The mechanism of antimicrobial action probably involves the disruption of the bacterial membrane, lysis of the bacteria, and leakage of intracellular contents. The inhibition of efflux pumps, prevention and destruction of existing biofilm, and inhibition of bacterial motility and membrane ATP-ases are also mentioned. Both compounds act additively or synergistically with conventional antibiotics, which is essential in overcoming the problem of bacterial resistance [39].

Oregano is a rich source of biologically active substances with antioxidant properties [40,41,42]. In 100 g of raw material, 1406–2221 mg of phenols, 4.2–23.1 mg of L-ascorbic acid, and 25.5–51.0 mg of carotenoids were found, with more phenolic compounds found in dry material and L-ascorbic acid and carotenoids in fresh material [43]. Oregano is a valuable source of rosmarinic acid (0.12–6.8%), a compound with valuable therapeutic properties [44,45]. Rosmarinic acid is the predominant phenolic compound of *O. vulgare*; its content in the extract from flowers is 0.99–9.65 mg·g^−1^, from leaves is 1.11–7.42 mg·g^−1^, and from stems is 0.53–0.77 mg·g^−1^ [40]. In addition, ursolic acid (3.80 mg·g^−1^), oleanolic acid (4.20 mg·g^−1^), β-sitosterol (5.40 mg·g^−1^), and triacontanol (6.12 mg·g^−1^) are present in the raw material of oregano [46]. The oregano herb also contains minerals, with higher amounts of calcium, potassium, magnesium, phosphorus, sulfur, and zinc [47], which, while playing a significant role in the normal functioning of the human body, may also be involved in the biosynthesis of the main components of the essential oil. Kanias et al. [48] showed a significant correlation between trace element content (Fe, Cr, Zn) and the concentration of carvacrol, thymol, and δ-cadinene, as well as chromium, iron, and thymol content and some micronutrients and carvacrol in OEO. The authors further suggest that higher iron and chromium concentrations increase the carvacrol content and decrease the proportion of thymol in OEO, while europium (Eu) may play a role in the metabolism of the taxon.

### 3.3. Biological Activity of Oregano (Selected Most Important Aspects)

#### 3.3.1. Antimicrobial and Antibiofilm Activity

The biological activities of *Origani herba* are broad, and one of the most potent is antimicrobial activity (Table 2). The active constituents of oregano have antiseptic, bactericidal, fungicidal, and virucidal effects [34,49,50], and perhaps most importantly, are effective in destroying biofilm [51]. The biofilm, which protects microorganisms by enabling them to survive in hostile environments and conferring antibiotic resistance, accounts for more than 80% of bacterial infections worldwide [52]. This complex multicellular structure of bacteria and fungi, surrounded by a layer of organic and inorganic substances produced by microorganisms, shows adhesion to biological and abiotic surfaces. Quorum sensing (QS), a bacterial communication mechanism using diffusible molecules called autoinducers, plays a vital role in the induction of gene expression to control cellular behaviors such as bioluminescence, the secretion of virulence factors, biofilm development, and survival against antimicrobials. QS and biofilm formation processes are interdependent [53]. Both Gram-positive and Gram-negative bacteria utilize QS [54]. Biofilm formation by pathogenic bacteria is considered a major virulence factor, protecting against unfavorable environmental conditions and host immune response mechanisms and against the targeted effects of antimicrobial agents. According to studies, 70% of all bacterial infections in humans are due to biofilm and lead to various diseases, including chronic wound healing [53]. Antibiotic therapy in patients colonized by *Pseudomonas aeruginosa* often relieves symptoms but does not cure the ongoing infection, as it cannot eliminate antibiotic-resistant biofilm communities [55]. Studies on biofilm-associated bacterial species have shown that quorum quenching products reduce antibiotic resistance in bacteria [53].

Many aromatic plants can inhibit biofilm formation mechanisms [52]. Monoterpenoids, among others, show the effects of antibiofilm on Gram-positive and Gram-negative bacteria in the first stages of biofilm development. The results of Upadhyay et al. [71] suggest that *trans*-cinnamaldehyde, carvacrol, thymol, and eugenol can potentially be used to control the biofilm of *Listeria monocytogenes*, a major foodborne pathogen. Cáceres et al. [72] showed that essential oils of the thymol–carvacrol chemotype from *Lippia origanoides* and *Thymus vulgaris*, with MIC values of 0.37–0.75 mg·mL^−1^, strongly inhibited biofilm formation and virolecin (QS) production in a concentration-dependent manner. According to the authors, the activity of thymol–carvacrol oils positions them as promising natural products in developing new and improved therapeutic strategies against bacterial resistance [72]. Proškovcová et al. [51], investigating the antibiofilm activity of Lamiaceae essential oils, proved that *O. vulgare* (0.1 mg·mL^−1^ and 0.3 mg·mL^−1^) and *T. vulgaris* (0.1 mg·mL^−1^ and 0.4 mg·mL^−1^) essential oils were the most effective in the adhesion and biofilm formation phase. The authors point to these products as potential antifungal treatments or prophylaxis agents that reduce pathogen resistance. Nostro et al. [56] showed that the susceptibility of methicillin-resistant strains of Staphylococcus depends on the OEO concentration and the thymol and carvacrol content. The increased proportion of these components in the oil may prove to be an element that potentiates its antimicrobial activity and antibiofilm activity. Studies of *P. aeruginosa* strains have shown that carvacrol and thymol interfere with the initial phases of adhesion and biofilm formation [73]. Khan et al. [33] report that carvacrol and thymol isolated from *O. vulgare* exhibit good bactericidal and antimicrobial activity against *Streptococcus mutans* and can be used as potential agents in controlling dental caries. Using the Gram-positive bacteria *S. aureus* and *S. epidermidis* as model organisms, Nostro et al. [56] investigated their ability to form a biofilm in the presence of OEO, thymol, and carvacrol. It was shown that bacteria cultured in the presence of subinhibitory concentrations of OEO and both compounds showed a reduced biofilm-forming capacity, with a more significant effect observed at higher concentrations.

OEO showed good antibacterial activity against multidrug-resistant bacteria and other types of bacteria [57]. OEO was active in inhibiting all microorganisms evaluated by Arámbula et al. [34]; however, it showed more significant activity in inhibiting the growth of *Escherichia coli* and *S. aureus* than *P. aeruginosa*. The authors suggest that the antibacterial activity of OEO provides an opportunity to develop a product for the pharmaceutical industry with inhibitory activity against bacteria such as *S. aureus*, *E. coli*, and *P. aeruginosa*. Similarly, Drăgan et al. [58] report that *P. aeruginosa* was the microorganism with the most outstanding resistance, while *S. aureus* proved to be the most susceptible pathogen to OEO, with medium inhibition. Marques et al. [59] confirmed the susceptibility of *S. aureus* to OEO. Simirgiotis et al. [60] showed that the pathogenic bacteria *S. aureus* and *Salmonella enterica*, as well as the phytopathogenic bacteria *Erwinia rhapontici* and *Xanthomonas campestris*, were the most sensitive to OEO, with the lowest concentration of oil required for their inhibition. The OEO tested contained thymol (15.9%), δ-terpinene (10.6%), p-cymene (8.6%), sabinene (6.5%), Z-sabinene hydrate (13.4%), linalyl acetate (7.2%), and carvacrol (3.1%). OEO further showed antimicrobial activity against bacteria associated with food poisoning. The authors point out that the higher antimicrobial activity of OEO may be explained by the higher amount of phenolic oxidized monoterpenes—especially thymol, p-cymene, and carvacrol, which may have had a synergistic effect causing destabilization of the cell membrane. According to our recent study [61], commercial OEOs containing high amounts of carvacrol (76.64–85.70%) exhibit antimicrobial activity. We particularly noted the excellent activity of one of the tested OEOs against two MRSA strains (ATCC 43300 and ATCC BAA-1707), which can be linked to carvacrol, terpinene, and β-bisabolene. Boughendjioua and Seridi [62] demonstrated that, despite the resistance of Gram-negative bacteria to multiple EOs, Bacillus strains showed sensitivity to EOs (p-cymene 24.01%, thymol 23.49%, and carvacrol 21.31%), with inhibition zones ranging from 21.5 mm (*B. amyloliquefaciens* S499) to 41 mm (*B. pumilus*). The MIC value obtained for all strains tested (0.4 mg mL^−1^) indicates the high activity of OEO against Bacillus. The high content of phenolic derivatives and effective antibacterial agents can explain this effectiveness. OEO also shows good bactericidal and antibiofilm activity against *S. mutans*, one of the essential bacteria causing tooth decay, and can be used as a preventive agent in the fight against this disease. The main components of OEO, thymol, and carvacrol induce autolysis, stress, and growth inhibition and reduce biofilm formation by *S. mutans* [33].

Carvacrol and thymol are phenols with potent antimicrobial activity and have been extensively studied for their ability to inhibit the proliferation of various bacteria. Both compounds are believed to have a similar individual spectrum of action on bacteria (Table 3) and exhibit a synergistic effect [74]. Among others, they have been shown to inhibit the growth of harmful organisms such as *E. coli*, *L. monocytogenes*, and *S. enterica* subsp. *enterica* serovar Typhimurium [39]. Kunz de Jesus et al. [75] describe the in vitro activity of carvacrol–thymol combinations with antibiotics (azithromycin, clarithromycin, minocycline, and tigecycline) and antifungal agents (amphotericin B, caspofungin, itraconazole, and terbinafine) against *Pythium insidiosum*. The authors concluded that combinations of carvacrol or thymol with these antimicrobials may provide effective alternative treatments for cutaneous pythiosis in animals due to their synergistic interactions. Magi et al. [76] showed that carvacrol works alone or in combination with erythromycin against cell-invasive, erythromycin-resistant group A streptococci and could be a novel therapeutic tool.

OEO is not only a potent bactericide but also an effective fungicide. Our study [61] proved that commercial high-carboxylic EOs showed antimicrobial activity, particularly against fungi (MIC = 0.06–0.25 mg·mL^−1^). *Candida parapsilosis* ATCC 22019 proved to be the most sensitive fungus against all EOs, with a value of MIC = 0.06 mg·mL^−1^. The EO containing the most carvacrol and o-cymene showed the highest activity (MIC = 0.06–0.125 mg·mL^−1^) against all Candida strains. The carvacrol content was negatively correlated with MIC values for three Candida strains (*C. lusitaniae* ATCC 34449, *C. albicans* ATCC 2091 and *C*. *albicans* ATCC 10231). OEO (thymol 27.3%, γ-terpinene 20.7% and carvacrol 16.1%) can be used as a fungicide against fungal infections caused by azole-resistant strains of *C. glabrata*. Combination therapy with standard antifungal drugs is suggested to avoid cytotoxic effects [81]. Baj et al. [49] found that OEO (carvacrol 57.3%, 1,8-cineole 12.9%, thymol 4.7%) showed activity against yeasts belonging to the reference strains *C. albicans* and *C. glabrata*, isolated from the oral cavity of different patient groups. OEO could inhibit the growth of pathogenic yeasts and kill them at the same or slightly higher concentrations. The experimental results of Lakhrissi et al. [63] show that the EOs of oregano, marjoram, and their combination have outstanding efficacy against *C. albicans*. The zone of inhibition is 30 mm for EO, 18 mm for marjoram, and 28 mm for the mixture. The antifungal activity of OEO may be due to the different chemical composition (quantitative and qualitative differences). Some authors report a synergistic effect of carvacrol and thymol and that p-cymene increases the antimicrobial activity of these compounds [64,81].

In addition to EOs, MAPs also provide hydrolates, valuable by-products of the oil industry. A hydrolate (hydrosol) is the distilled aromatic water remaining after aqueous or steam distillation and EO separation [82]. Hydrolates are produced through steam distillation using the same isolation process as EO. During industrial distillation, water evaporates simultaneously with EOs, and after vapor condensation, the components are separated into two phases in a collection vessel: EO and hydrolate. A small amount of EO components remains in the hydrolate, providing specific organoleptic and taste properties and biological activity [83,84]. The inhibitory effect of *O. vulgare* hydrolates against *E. coli* and *S. aureus* allows for the decontamination of foodborne pathogens from freshly cut vegetables. *S. aureus* was found to be sensitive to hydrosol treatments, which completely eliminated this pathogen after 40 min [85].

OEX, which contain other polyphenolic constituents, show varying antimicrobial activity (Table 2). Teixeira et al. [65] report that ethanolic OEX and OEO have antimicrobial properties, which are more potent for oil. A study by Coccimiglio et al. [66] showed that ethanolic OEX has strong cytotoxic, antioxidant, and antimicrobial activity, which is mainly attributed to the presence of carvacrol (59.46%) and thymol (25.00%). The authors demonstrated that ethanolic OEX can inhibit non-mucous and mucous clinical isolates of *P. aeruginosa* and clinical isolates of *B. cenocepacia*. The Gram-negative pathogens are typical opportunistic bacilli in our environment that cause infections in patients with lung diseases such as cystic fibrosis. Bankova and Popova [67] report that oregano infusion has potent in vitro antibacterial and antifungal activity, similar to thiamphenicol, a broad-spectrum control antibiotic. The tested strains of *P. aeruginosa* and *C. albicans* showed the highest sensitivity, and *P. multocida* the lowest. Cold water extract showed weaker antimicrobial activity, pronounced against *P. aeruginosa* and *K. pneumoniae* strains, but not against *P. multocida* strains. In contrast, the hot water extract had the weakest in vitro antimicrobial activity. Brđanin et al. [68] proved more significant activity of OEX against bacteria, especially Gram-positive bacteria, than against fungi. The cyclohexane extract of *O. vulgare* showed no activity against *Helicobacter pylori*, while the dichloromethane and methanol extracts were active against *S. typhimurium*. As the main phenolic compound in the methanolic OEX, rosmarinic acid may be involved in the activity shown.

Rahmani Gohar et al. [69], in a rat model, showed that the topical application of OEX (alcohol macerate) to infected wounds had significant antimicrobial activity against *S. aureus* (ATCC 29213). OEX showed antimicrobial activity against *S. aureus*, *E. coli*, and *C. albicans*. In addition, the most significant effect was achieved against a Gram-positive bacterium (*S. aureus*); inhibitory effects on Gram-negative bacteria and fungi were also noted, but at higher extract concentrations [70]. Similarly, Bešta-Gajević et al. [86] showed that Gram-positive bacteria (*Bacillus subtilis*, *S. aureus*, and *S. aureus*—MRSA) were more sensitive to oregano leaf and flower extracts than Gram-negative bacteria. Gram-negative bacteria were differentially sensitive to methanolic extracts but insensitive to aqueous extracts. *C. albicans* proved resistant to aqueous and methanolic OEX.

#### 3.3.2. Antioxidant Activity

Most human diseases are associated with the excessive accumulation of reactive oxygen species produced during immune system activity or induced by external factors. EOs are known for their natural antioxidant activity [87]. The antioxidant activity of EOs depends on the presence of metabolites containing conjugated double bonds or phenolic groups [4]. Phenolic antioxidants can inhibit free radical formation, interrupt the propagation of autoxidation, or both. Thymol and carvacrol are electron-delocalized systems due to their hydroxyl, methyl, and isopropyl groups. Therefore, they serve as hydrogen or electron donors, allowing the transformation of the DPPH-radical to its reduced form DPPH-H and the reduction of Fe^3+^ [88].

Stanojević et al. [89], when testing an OEO rich in thymol (45%) and carvacrol (37.4%), found that OEO incubated for 60 min showed the best antioxidant activity. The concentration of OEO required to neutralize 50% of the initial DPPH radical concentration (EC_50_) was 0.761, 0.590, 0.360, and 0.326 mg·mL^−1^ after 20, 30, 45, and 60 min of incubation, respectively. Lipid peroxidation inhibition of 92.3% was achieved at an OEO concentration of 1.35 mg·mL^−1^. Similarly, Drăgan et al. [58] demonstrate that OEO exhibits antioxidant efficacy, as indicated by stable DPPH free radical scavenging factor values (80.80–68.47 μg·mL^−1^). Our study [61] demonstrates that the antioxidant activity of OEO, as determined by the DPPH assay, is high (71.4–80.4%) relative to the control (1% vitamin C—90%). Previously, Jianu et al. [36] showed that *O. vulgare* var. *aureum* EO had better uptake activity in DPPH and ABTS assays than ascorbic acid. In the beta-carotene/linoleic acid bleaching test, the EO showed higher relative antioxidant activity, but lower than that of BHA. The authors suggest that p-cymene and γ-terpinene may contribute to OEO’s biological antioxidant activity by inhibiting reactive oxygen species (ROS)-producing enzymes such as lipoxygenase and xanthine oxidase. The results indicate that OEO is a good source of natural antioxidants with potential applications in the food and pharmaceutical industries and a safer alternative to synthetic antioxidants.

Regarding non-volatile constituents, OEX has the most effective antioxidant activity among aromatic herbs [90]. Teixeira et al. [65] suggest that hot-water OEX has strong antioxidant properties. Benchikha et al. [42] demonstrated that ethanolic OEX and OEO exhibit antioxidant activity compared to standard antioxidant compounds (BHA and α-tocopherol) and can be used as an available source of natural antioxidants. Research by Rosmalena et al. [91] indicates that ethanolic OEX from stem bark containing saponins, flavonoids, alkaloids, terpenoids, steroids, essential oil, and tannins shows moderate antioxidant activity in reducing DPPH radical levels in vitro, weaker than vitamin C. The antioxidant activity of OEX was further investigated in vivo by comparing malondialdehyde (MDA) levels. MDA is formed by the long-chain peroxidation of unsaturated fatty acids in fatty membranes, which occurs under oxidative stress. Mean MDA levels decreased after treatment with oregano extract (0.761 ± 0.107 and 132 ± 0.168 nmol·mL^−1^, respectively). The authors considered OEX a potential herbal antioxidant due to its low IC_50_ and effectiveness in reducing MDA levels.

The antioxidant activity of plants is positively correlated with the total phenolic, flavonoid, and flavanol content [6]. Kaurinovic et al. [92] report that different extracts (EtOAc, *n*-BuOH and H_2_O) from wild-grown oregano in Serbia inhibited the OH^•^ radical. The EtOAc extract, distinguished by having the highest content of phenolic compounds and flavonoids, showed the highest inhibitory activity. The authors further suggest that the polarity of the flavonoid components influences their ability to inhibit lipid peroxidation in liposomes. Strong antioxidant properties were demonstrated by a methanolic extract from fresh oregano herb, in which rosemary and protocatechuic and caffeic acids were determined [45]. Similarly, Bešta-Gajević et al. [86] showed the intense antioxidant activity of methanolic extracts from oregano leaves, while aqueous extracts had low antioxidant potential (36.63% for flower extracts and 29.54% for leaf extracts). In contrast, Rodriguez et al. [93] demonstrated the antioxidant activity of a dry oregano herb extract containing, in addition to phenolic compounds, other components structured as flavonones, dihydroflavonols, flavonols, and flavones. Capecka et al. [43] report that fresh and dried herbs of oregano and other plants of the Lamiaceae family are rich sources of antioxidants, especially from the phenolic compound group. The antioxidant activity of OEX appears to contribute to its prophylactic effect against inflammatory diseases, especially those with stress-related causes [94]. These findings are relevant given the widespread use of oregano as a spice.

The antioxidant activity of oregano offers great potential for use in prevention and treatment. However, gastrointestinal digestion may modify some therapeutic properties after oral administration. To avoid this, different solid oral formulations for *O. vulgare* extract are being analyzed, considering their in vitro and in vivo antioxidant activity after simulated gastrointestinal digestion. De Torre et al. [95] propose an encapsulated form, including two capsules containing 250 mg of aqueous *O. vulgare* extract, with a minimum of 33% rosmarinic acid, as a daily dose.

#### 3.3.3. Antiangiogenic and Antitumor Potential

Angiogenesis is a standard and tightly controlled physiological process defined as the formation of new blood vessels necessary for specific physiological and pathological states, including tumor growth, development, and metastasis. This process is also involved in an essential step in the transition of a tumor from a dormant to a malignant state. Therefore, angiogenesis inhibitors are used in the treatment of cancer [3]. Angiogenesis inhibition and other anticancer strategies, such as chemotherapy, appear invaluable in achieving optimal outcomes in cancer patients. Some natural plant-derived compounds can prevent the formation of new blood vessels in the tumor and inhibit the proliferation and growth of cancer cells by inhibiting the function of the cyclooxygenase (COX) enzyme, one of the most critical active enzymes in the angiogenesis pathway [96,97]. Carvacrol has potent anti-inflammatory effects by inhibiting the COX-2 enzyme and plays a role in reducing oxidative stress. By activating apoptosis, this compound has an antiproliferative effect on lung and breast cancer cells and significantly inhibits cell migration and angiogenesis. However, there is also evidence for the induction of angiogenesis by carvacrol. This inconsistency may be due to the dose of carvacrol used [97].

Some EOs are non-toxic antiangiogenic agents and may have potential therapeutic applications. Bonstancioglu et al. [98] described the ability of EOs to inhibit tumor cell viability and angiogenesis. It has also been reported that carvacrol prevents lipid peroxidation and liver cell damage and protects the antioxidant system in DEN-induced hepatocellular carcinogenesis [99]. Jaafari et al. [100] showed that thymol and carvacrol have cytotoxic effects on cancer cell lines, with carvacrol proving to be the more cytotoxic compound. Carvacrol and carveol were further shown to arrest cell cycle progression in the S phase; however, thymol and isopulegol arrested it in the G0/G1 phase. These findings suggest a proapoptotic effect of carvacrol, the main bioactive compound with proapoptotic and antiangiogenic effects [3].

OEO can serve as a cytotoxic agent against gastric cancer cell lines: it induces apoptosis and inhibits invasion and migration. OEO treatment can also inhibit lipogenesis and cholesterol biosynthesis at the molecular level [101]. OEO showed significant activity against cancer cell lines A549 (IC_50_, 27.2 µg·mL^−1^), Hep3B (IC_50_, 7.4 µg·mL^−1^) and MCF-7 (IC_50_, 7.1 µg·mL^−1^) compared to the standards. In addition, aqueous OEX showed exceptional activity against Hep3B (IC_50_, 27.2 µg·mL^−1^) and MCF-7 (IC_50_, 10.8 µg·mL^−1^) cell lines. The activity of the essential oil may be due to the presence of carvacrol, as this compound is the main component of the essential oil, with a high percentage (90.4%), or to the synergistic action of the compounds contained in the essential oil [102]. Bouhtit et al. [103] demonstrated that combining carvacrol and thymol induced cancer cell death with low toxicity to normal cells. The authors highlighted that various molecular pathways are involved in this potential synergistic effect, including apoptosis, oxidative stress, reticular stress, autophagy, and necrosis. OEO shows promising anticancer activity, but a detailed study is warranted to confirm how the apoptosis pathway and the lipid biosynthesis pathway interact to induce cancer cell death.

In the report prepared by EFSA [104], a scientific opinion was presented on the use of oregano and lemon balm extracts as food additives. It was stated that there is limited characterization of the active compounds present in the extracts, especially phenols, and therefore, this is a so-called presumed safety, which may not apply to specific cases. The report also indicates that oregano extracts are considered natural extracts and generally recognized as safe. However, the application proposed a phenol intake of 2.0 mg/kg bw/day for women and 2.3 mg/kg bw/day for men, which the committee considered to be within the limits of safe phenol consumption; however, due to the lack of precise characterization and specification of the extracts and the lack of data on the genotoxicity, reproductive and developmental toxicity, and long-term toxicity of oregano extracts, the safety of extracts obtained from oregano cannot be assessed.

## 4. Conclusions

The results of the studies indicate that oregano is a precious spice and medicinal plant. The oregano herb contains extremely active compounds with antimicrobial and antioxidant properties (OEOs, polyphenols, pigments) and may be a valuable source of natural antiseptic and protective agents.

When analyzing the usefulness of OEOs in prevention and treatment, attention should be paid to the phenolic compounds thymol and carvacrol, which exhibit significant antioxidant, anti-inflammatory, antiangiogenic, anticancer, and antimicrobial activities. The findings highlight the promising role of carvacrol and thymol as potential structures in the search for new antimicrobial and antibiofilm agents. Biofilm infections are a severe problem due to their high resistance to available antimicrobial drugs. Other components of OEO are also worthy of consideration due to their activity and possible synergistic effects.

The rich and varied chemical profile and the unique biological activity of oregano are the basis for further research toward evaluating biochemical variability, the mechanisms of action of individual compounds and extracts, and possible combined applications with other preparations. An important direction of future research seems to be the analysis of the chemical composition of oregano products offered by manufacturers (essential oils, supplements, and other products) in order to examine their effectiveness and safety, as well as to create recommendations for use.

## Figures and Tables

**Table 1:** Diversity of dominant components of *O. vulgare* essential oil (OEO).

Species	Origin	Dominant Components of OEO	Contents (%)	References
*O. vulgare*	Macedonia	α-pinene	40.0–67.5	[29]
		β-caryophyllene	7.4–26.6	
*O. vulgare*	Syria	terpinen-4-ol	24.9	[32]
		γ-terpinene	10.6	
		*o*-cymene	8.9	
		*cis*-β-terpineol	8.7	
*O. vulgare*	Saudi Arabia	carvacrol	80	[33]
*O. vulgare*	Jordan	thymol	69	[33]
*O. vulgare*	Colombia	thymol	30.6	[34]
		thymol methyl ether	17.4	
		α-terpinene	15.7	
		carvacrol	8.1	
*O. vulgare* ssp. *vulgare*	Lithuania	thymol	39.3	[25]
		terpinen-4-ol	≤39.5	
		sabinene	≤40.1	
		*cis*-sabinene hydrate	≤41.2	
*O. vulgare* ssp. *vulgare*	Turkey	caryophyllene	14.4	[35]
		spathulenol	11.6	
		germacrene D	8.1	
		α-terpineol	7.5	
*O. vulgare* ssp. *vulgare*	Estonia	linalool	0.3–20.6	[26]
		β-caryophyllene	1.3–45.0	
		germacrene D	0.7–21.0	
		caryophyllene oxide	1.5–31.3	
		spathulenol	0.9–10.1	
*O. vulgare* ssp. *vulgare*	Iran	carvacrol	6.4–21.3	[31]
		sabinene	0.9–20.8	
		γ-terpinene	7.1–17.5	
		*trans*-caryophyllene	1.2–11.3	
		(E)-β-ocimene	1.6–10.4	
*O. vulgare* ssp. *vulgare*	Montenegro	germacrene D	15.4–27.9	[30]
		β-caryophyllene	7.7–14.6	
		*α*-terpineol	4.8–17.8	
		linalyl acetate	0.5–9.6	
		linalool	3.0–8.8	
		thymol	0.2–8.3	
		terpinene 4-ol	1.5–8.3	
*O. vulgare* ssp. *hirtum*	Montenegro	carvacrol	74.3	[30]
*O. vulgare* var. *aureum* L.	Romania	γ-terpinene	22.9	[36]
		p-cymene	14.7	
		germcrene D	11.6	

**Table 2:** Antimicrobial activity of *O. vulgare* L.

Essential Oil/Extract	Origin	Type of Microorganism	MIC (mg·mL^−1^)	Reference
EO	Slovakia	*Candida albicans*	0.4	[51]
EO of aerial parts	Italy	Methicillin-susceptible and methicillin-resistant *Staphylococci* (MSS, MRS)	0.06–0.125% (*v*/*v*)	[56]
EO Aerial dried parts	Iraq	*Escherichia coli* multidrug resistance *K. pneumoniae* *Staphylococcus aureus S. pneumonia;* *Enterococcus faecalis*	-	[57]
EO	municipalities of Pamplona and Ragonvalia, Colombia	*E. coli* *S. aureus* *P. aeruginosa*	15.62	[34]
			15.62	
			62.5–125	
EO	Romania	*S. aureus* ATCC 25923 *E. coli* ATCC 25922	an average inhibition varying between 19.67 mm for the 1:1 diluted oregano oil samples and 35.67 mm for the concentrated oregano oil samples	[58]
EO	-	*S. aureus*	-	[59]
EO	arid Andean region	*E. coli* *P. aeruginosa* *Salmonella* *enterica* *Bacillus subtilis* *S. aureus*	0.04% *v*/*v*–0.63% (*v*/*v)*	[60]
EO	Commercial	*C. albicans* ATCC 10231 *C. albicans* ATCC 2091 *C. auris* CDC B11903 *C. glabrata* ATCC 90030 *C. krusei* ATCC 14243 *C. parapsilosis* ATCC 22019 *C. lusitaniae* ATCC 34449 *C. tropicalis* ATCC 1369	0.06–0.25	[61]
		Methicillin-resistant *S. aureus*: *S. aureus* ATCC 43300; *S. aureus* ATCC BAA-1707	0.25–0.5	
		*Micrococcus luteus* ATCC 10240 *B. subtilis* ATCC 6633 *Bacillus cereus* ATCC 10876	0.125–0.25	
		*B. bronchiseptica* ATCC 4617	0.06–0.125	
EO fresh herbs, aerial part of the plant	Northeast of Algeria	*B. amyloliquefaciens* FZB42 *B. amyloliquefaciens* S499 *B. subtilis* ATCC 21332 *B. licheniformis* ATCC 14580 *B. pumilus*	0.4	[62]
EO herbs	Poland	*C. albicans* *C. glabrata*	250–500	[49]
EO herbs	Morocco	*C. albicans*	1/100 (*v*/*vvv*)	[63]
EO	Commercial product from pharmacies	*S. aureus* *Listeria monocytogenes*	50	[64]
		*C. albicans* *Enterobacteraerogenes* *B. subtilis * *S. typhymurium* *C. glabrata*	25	
		*Aspergillus niger Penicillium claviforme* *Saccharomyces cerevisae* *E. coli*	12.5	
**Extracts**				
Ethanolic extracts	Portugal	*S.* Typhimurium	13.9	[65]
		*E. coli*	6.9	
		*Lippia origanoides*	13.9	
		*Listeria monocytogenes*	6.9	
		*Shewanella putrefaciens*	277	
		*Brochothrix thermosphacta*	277	
Ethanolic extracts	Greece	*P. aeruginosa*	6.3–25	[66]
		*Bordetella bronchiseptica*	12.5	
		*E. coli*	12.5–25	
		*Burkholderia cenocepacia*	6.3–25	
		*Acinetobacter woffii* *A. baumannii* *Moraxella catarrhalis*	12.5	
		*B. subtilis*	6.3	
		*S. aureus*	12.5–25	
Hot aqueous extracts (infusion); loose dry leaves and flowers	Bulgaria	*P. aeruginosa* *C. albicans* *Pasteurella multocida*	-	[67]
Cold aqueous extracts; loose dry leaves and flowers	Bulgaria	*Klebsiella pneumoniae*		
Dichloromethane and methanol extracts; the air-dried, powdered aerial parts	Serbia	*Helicobacter pylori* *C. albicans*	0.125–0.25	[68]
Alcohol macerate; Leaves	Iran	*S. aureus* ATCC 29213 *E. coli* *C. albicans*	-	[69]
Supercritical Fluid Extraction (SFE), leaf and flower extracts	Wild oregano (*O. vulgare*) purchased from Alfred Galke GmbH (Samtgemeinde Bad Grund, Germany)	*B. subtilis* *S. aureus*—MRSA	-	[70]

**Table 3:** Antimicrobial activity of thymol and carvacrol.

Component	Bacteria	Source of Information
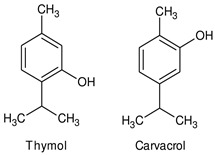	*P. aeruginosa*, *S. aureus*, *S. mutans*, *S. epidermidis*, *Enterobacter aerogenes*, *E. coli*, *L. monocytogenes*, *B. subtilis*, *S.* Typhimurium, *Clostridium perfringens*, *Salmonella* strains	[33,39,73,74,77,78,79,80]

## Data Availability

Data are contained within the article and Supplementary Materials.

## Supplementary Materials

The following supporting information can be downloaded at https://www.mdpi.com/article/10.3390/ph18020267/s1. Photo S1: *Origanum vulgare* L. morphology; Photo S2: Oregano plants in full bloom.
