# Supplementary figures and images for: Chemical Composition, Biological Activity, and Potential Uses of Oregano (Origanum vulgare L.) and Oregano Essential Oil
Source: Pharmaceuticals (Basel). 2025 Feb 18;18(2):267. doi: 10.3390/ph18020267 (PMC11858988; doi:10.3390/ph18020267)

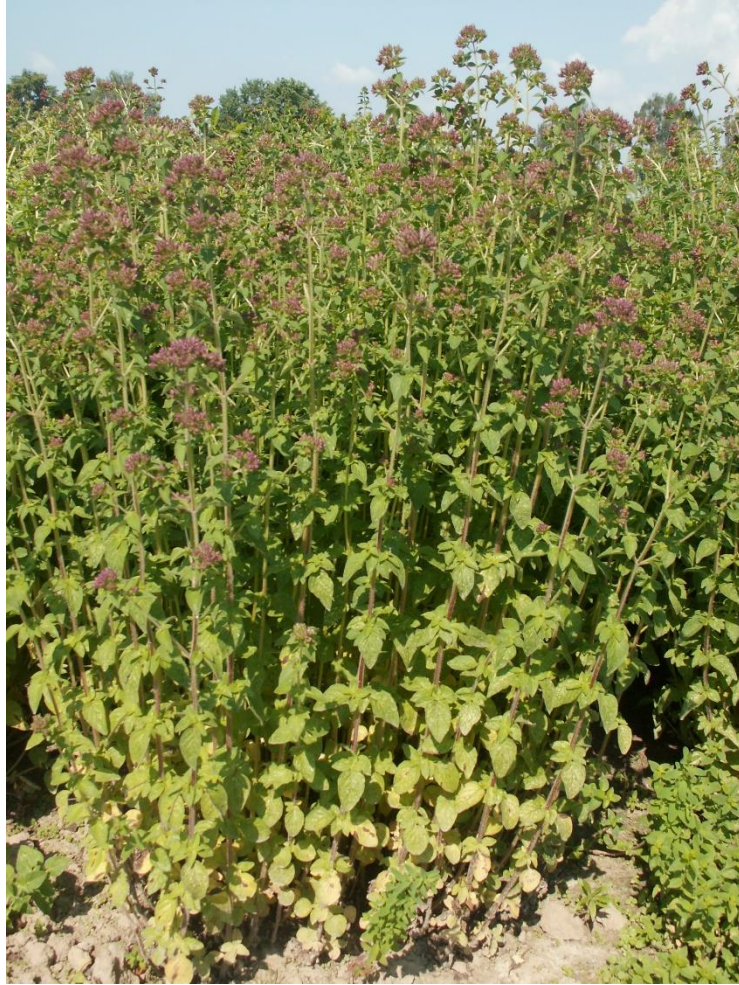

Photo S1. *Origanum vulgare* L. morphology.

Supplement: Supplementary file 1 [file pharmaceuticals-18-00267-s001.zip › Photo S1.pdf]

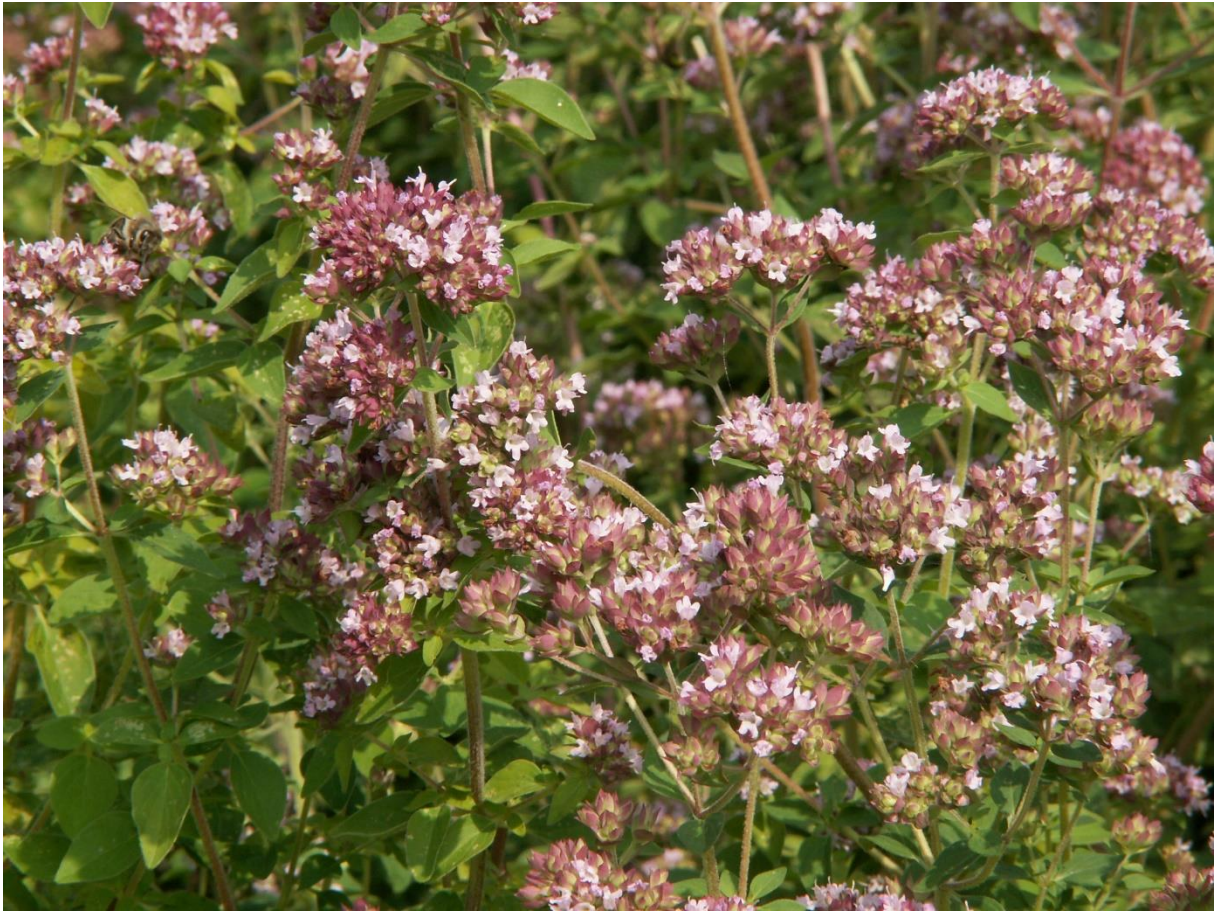

Photo S2. Oregano plants in full blooming.

Supplement: Supplementary file 1 [file pharmaceuticals-18-00267-s001.zip › Photo S2.pdf]
